# Replace and repair: Biomimetic bioprinting for effective muscle engineering

**DOI:** 10.1063/5.0040764

**Published:** 2021-07-08

**Authors:** Cooper Blake, Oliver Massey, Mitchell Boyd-Moss, Kate Firipis, Aaqil Rifai, Stephanie Franks, Anita Quigley, Robert Kapsa, David R. Nisbet, Richard J. Williams

**Affiliations:** 1 Institute of Mental and Physical Health and Clinical Translation, School of Medicine, Deakin University, Waurn Ponds, VIC 3216, Australia; 2 Biofab3D, Aikenhead Centre for Medical Discovery, St Vincent's Hospital Melbourne, Fitzroy, VIC 3065, Australia; 3 Biomedical and Electrical Engineering, School of Engineering, RMIT University, Melbourne, VIC 3000, Australia; 4 Laboratory of Advanced Biomaterials, The Australian National University, Canberra, ACT 2601, Australia

## Abstract

The debilitating effects of muscle damage, either through ischemic injury or volumetric muscle loss (VML), can have significant impacts on patients, and yet there are few effective treatments. This challenge arises when function is degraded due to significant amounts of skeletal muscle loss, beyond the regenerative ability of endogenous repair mechanisms. Currently available surgical interventions for VML are quite invasive and cannot typically restore function adequately. In response to this, many new bioengineering studies implicate 3D bioprinting as a viable option. Bioprinting for VML repair includes three distinct phases: printing and seeding, growth and maturation, and implantation and application. Although this 3D bioprinting technology has existed for several decades, the advent of more advanced and novel printing techniques has brought us closer to clinical applications. Recent studies have overcome previous limitations in diffusion distance with novel microchannel construct architectures and improved myotubule alignment with highly biomimetic nanostructures. These structures may also enhance angiogenic and nervous ingrowth post-implantation, though further research to improve these parameters has been limited. Inclusion of neural cells has also shown to improve myoblast maturation and development of neuromuscular junctions, bringing us one step closer to functional, implantable skeletal muscle constructs. Given the current state of skeletal muscle 3D bioprinting, the most pressing future avenues of research include furthering our understanding of the physical and biochemical mechanisms of myotube development and expanding our control over macroscopic and microscopic construct structures. Further to this, current investigation needs to be expanded from immunocompromised rodent and murine myoblast models to more clinically applicable human cell lines as we move closer to viable therapeutic implementation.

## I. INTRODUCTION

Volumetric muscle loss (VML), defined as the loss of more than 20% of skeletal muscle, destroys innate repair mechanisms and renders muscle tissue incapable of self-healing.^1^ Deleterious loss of muscle volume can result from external physical trauma or surgical excision due to cancer or ischemic necrosis. Typically, small injuries are repaired through macrophage phagocytosis of dead myofibers, followed by proliferation of satellite cells to repopulate the tissue. However, it is believed that the loss of local satellite cell populations and basal lamina in VML injuries prevents endogenous mechanisms from replacing lost tissue.^2^ VML injuries frequently suffer from a loss of vascularization, resulting in ischemia, which favors the formation of fibrotic tissue and limits functional restoration.^3^ Current treatments typically involve debridement of fibrotic tissue and insertion of autologous muscle grafts to promote muscle repair, followed by physical therapy. As with any surgery, this method often results in injurious inflammatory responses.^3^ VML has a considerable effect on the quality of life, with significant economic burden placed on both individuals and the healthcare system.^4^

**FIG. 1.**
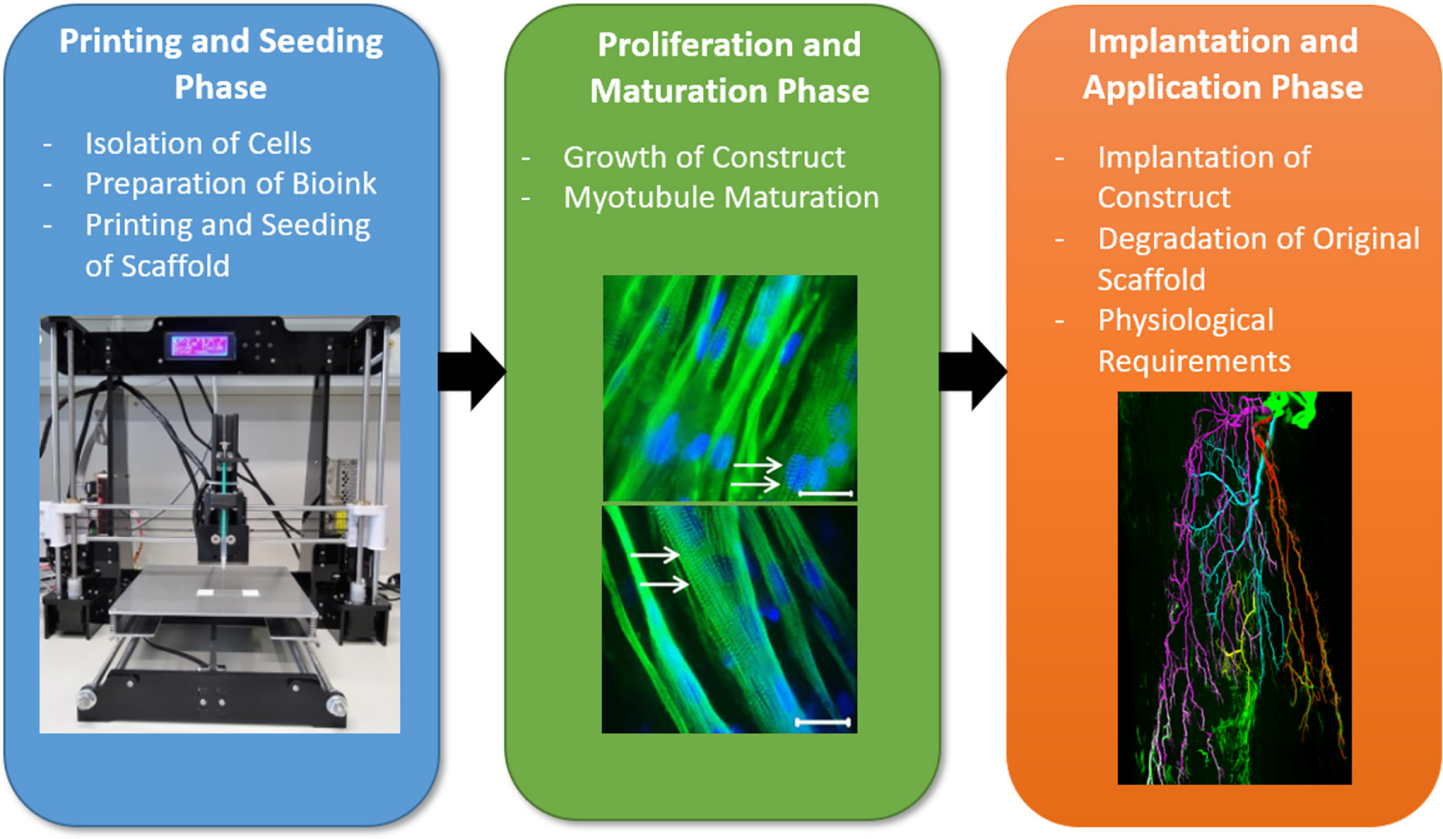
Summary of the phases of muscle biofabrication. Creation of mature muscle constructs requires three phases: (1) printing and seeding, including material preparation, cell isolation, and fabrication process; (2) growth and maturation, demonstrated by myotube multinucleation and formation of contractile striations; and (3) application and implantation into the host, with an image depicting the nerve fibers associated with muscles. All three phases are essential and pose unique challenges for biofabrication scientists. Images adapted and reproduced with permission from (1) Kahl *et al.*, Front. Bioeng. Biotechnol. **7**, 184 (2019). Copyright 2019 Authors, licensed under a Creative Commons Attribution (CC BY) license.^11^ (2) Chaturvedi *et al.*, PLoS One **10**(6), e0127675 (2015). Copyright 2015 Authors, licensed under a Creative Commons Attribution (CC BY) license.^12^ (3) Li *et al.*, Neurophotonics **7**(1), 015003 (2020). Copyright 2020 Authors, licensed under a Creative Commons Attribution (CC BY) license.^13^

As shown in Fig. 1, Novel bioengineering techniques, specifically, additive manufacturing, are therefore being explored as viable alternatives to traditional VML treatments. 3D bioprinting offers the chance to produce complex tissue constructs *ex vivo*, as a minimally invasive alternative to autologous muscle grafts. The development of bioprinted tissue engineered muscle aims to recreate the native structure and function of muscle by controlled deposition or patterning of biomimetic materials.^5–7^ *In vivo*, muscles are comprised of connective tissue, myofibers, satellite, and supporting cells, a vascular network and innervation, all of which function synergistically.^12^ Fabricating synthetic versions of complex muscle structures have been supported by recent advances in biofabrication technologies, with multiscale strategies allowing for biomimicry at both bulk (additive manufacturing) and cell scales (biomimetic material design). Bulk microchannel incorporation and topographical patterning have been achieved via methods of sacrificial layers or lyophilization and polymer or gold nanowire alignment. Aided by 3D biofabrication, complex muscle structures can develop vasculature and demonstrate maturing aligned myofibers supported by microchannel and topographical structures previously unavailable in 2D systems. Additional advances in biomimetic materials such as decellularized skeletal muscle matrix provide appropriate composition for muscle maturation.^126^ Together, translational success of biofabricated items, such as custom 3D-printed ankle and spinal implants,^8,9^ and regenerative hydrogels,^10^ demonstrates the possibilities for biofabrication to improve clinical outcomes. However, this success is yet to reach patients for VML. Two important considerations for skeletal muscle construct upscaling and translation to human subjects include angiogenesis, to improve nutrient diffusion and removal of metabolic waste, and innervation, for restoration of muscle control and function, thus facilitating production of larger constructs.

The array of bioinks, printing technologies, and techniques surrounding construct implantation and survivability have massively expanded in recent years, offering new opportunities to fine-tune construct biochemical properties, creating improved patient outcomes. Additionally, advancements in our understanding of the roles of tissue architecture, and endogenous physical mediators of tissue repair, have vastly improved myotubule alignment, vascularization, and innervation in transplanted constructs, which are vital for construct function and survival. 3D bioprinting for VML repair is a complex multidisciplinary process, composed of three key phases: printing and seeding, growth and maturation, and implantation and application. Each phase has its own unique challenges and considerations, and the key components of these phases are demonstrated in Fig. 1.

This review will highlight and describe the main techniques and processes involved in the various stages of bioprinting from initial construct creation to eventual clinical application, with a focus on recent successes (2015–2020 inclusive). Although there is currently limited evidence of human trials, recent successes in animal models hold much promise for the future of VML treatment. Importantly, every new study also elucidates the current limitations and gaps in knowledge, paving the way for future studies and strengthening our current understanding of the biochemical processes underpinning muscle tissue development and restoration.

## II. PRINTING AND SEEDING CONSTRUCTS

### A. Biofabrication

**FIG. 2.**
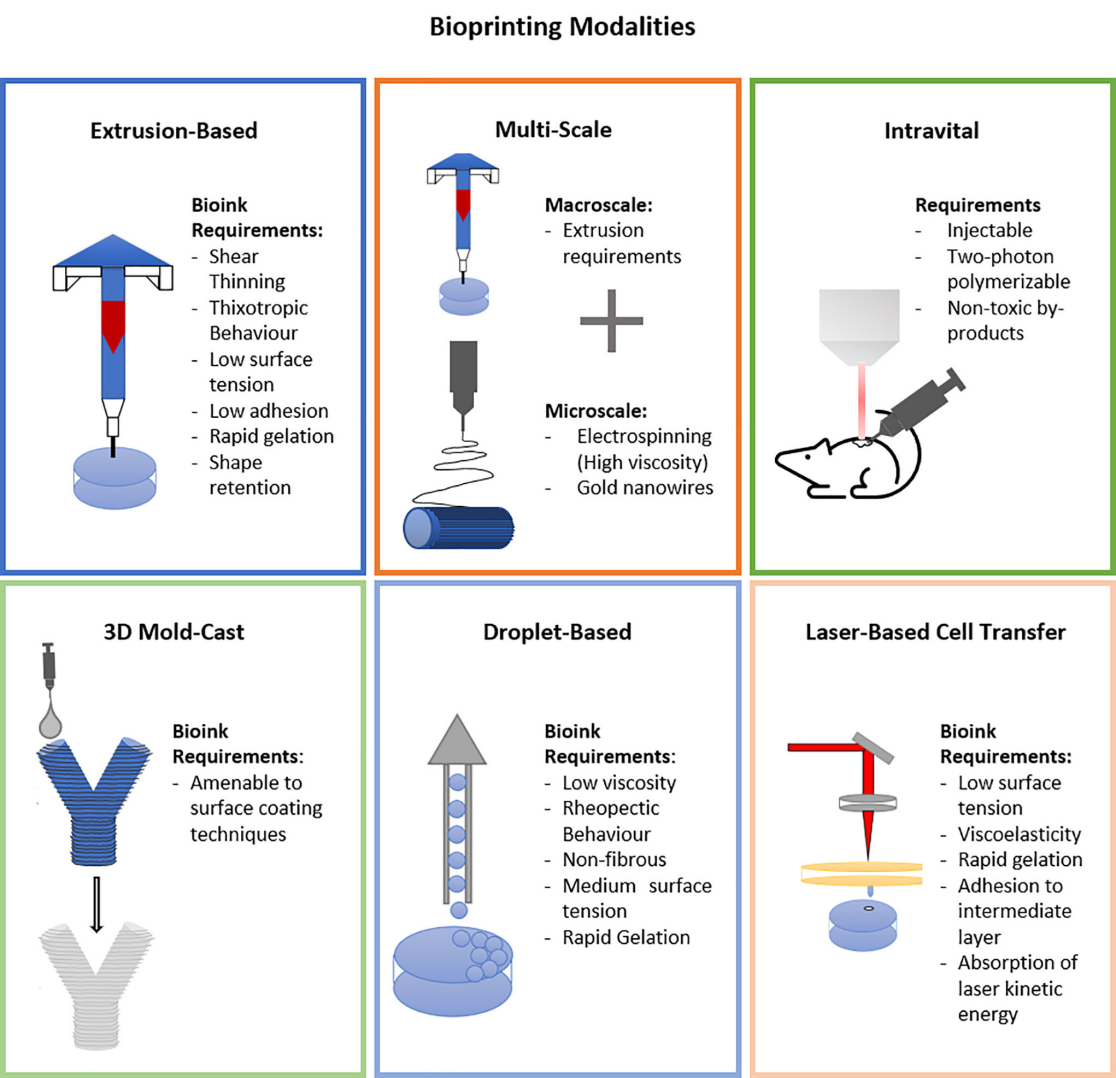
Bioprinting modalities: recent advances in muscle tissue engineering use several fabrication techniques, namely, extrusion printing, multiscale, intravital, 3D mold-cast, droplet-based and laser-based cell transfer, each of which require specific biomaterial properties.

Biofabrication of physiologically relevant muscle constructs requires structural control across multiple scales. Here, additive manufacturing techniques can be adapted^11^ to provides two major benefits: one, directional cues for aligned mature muscle formation and two, deposition into biomimetic and patient-specific structures. Recent advances in biofabricated muscle use a number of different mechanisms, as shown in Fig. 2, to fabricate 3D constructs. Primarily these include molding and casting,^14–16^ extrusion printing,^17–27^ multiscale fabrication,^28–34^ freeform fabrication,^35^ multimaterial extrusion printing,^36–39^ 3D mold-cast,^40^ layered cell-sheet techniques,^41,42^ wet spun fibers,^43^ and *in vivo* techniques such as 3D scaffold coating^44^ and intravital printing.^45^

#### 1. Micropatterning

Micropatterning directs cells into an aligned formation and is achievable with standard lithography methods, ultrasonic or magnetic fields, or extrusion-induced grooves. Recent studies have demonstrated that micropatterning lithography techniques can manipulate the microenvironment of tissue constructs including fibroblasts and human umbilical vein endothelial cells (HUVECs).^46^ However, while lithography shows promise for directing alignment, it relies on photomasks and molds and subsequently is limited to low complexity in 3D. As such, the resulting constructs cannot accurately facilitate *in vivo* cell behavior or structure, meaning lithography is ill-suited to the manufacture of complex 3D skeletal muscle constructs. For this reason, lithography will not be discussed in detail in this review.

Another micropatterning approach found that subjecting C2C12 myoblast-laden hydrogels to ultrasonic standing waves caused accumulation at static pressure nodes and the formation of highly aligned myotubules.^47^ De France *et al.* demonstrated that manipulation of anisotropic hydrogel-embedded cellulose nanocrystals with a magnetic field could induce alignment and differentiation of C2C12 myoblasts.^48^ Alternatively, microtopographical channels on the surface of extruded gelatin methacryloyl (GelMA) fibers have also proven capable of orienting C2C12 myoblasts seeded to the surface and inducing myotube development parallel to the direction of the channel cue.^49^ These micropatterning methods, in combination with macroscale biofabrication techniques, could provide important alignment cues and patient-specific shaped scaffolds.

Several groups have introduced topographical features via the molding and casting of 3D pillars. This technique promotes alignment of muscle cells via tensional force produced as the scaffold material contracts around two pillars.^14–16^ Cast hydrogels in the molds create pillars providing directional support for myotube formation. Molding and casting offer ease of fabrication with tension-supported alignment. However, pillar size has been shown to influence contractile models, with research suggesting two distinct modes of pillar displacement resulting in distinctly different cellular responses.^50^ Additionally, for applications requiring clinically relevant volumes of tissue-engineered muscle, further control into the third dimension would be required for microchannel diffusion of nutrients, signaling molecules and waste.

#### 2. Extrusion bioprinting

Significant interest has focused on extrusion printing as it provides control of structural features in three-dimensions on a relevant scale for muscle. Extrusion bioprinting is the controlled layer-by-layer deposition of materials, cells, or a combination of the two. Generally, extrusion printers employ pneumatic-, screw-, or piston-driven print-head that attach to a cartridge filled with the deposition material and a nozzle to control the extruded filament diameter. User-generated or computer-aided designs are fed into the axis's controller of the print head, allowing controlled deposition and patterning in three dimensions. Additional material-specific requirements can include thermal control of the print-head or platform, built-in UV light, or custom print-heads to aid material extrusion and retainment of post-extrusion shape. Recent works in muscle biofabrication via extrusion printing use a broad range of materials across classes of synthetic,^20^ semi-synthetic,^17^ natural,^19,21,22,24,26,34^ and blended-type materials^18,19,23,27^ (Table I).

**TABLE I.** Materials and alignment methods of recent advances in extrusion printed muscle constructs.

Material type	Material/s	Alignment method	Cell type	References
Synthetic	Polylactic acid (PLA), acrylonitrile butadiene styrene (ABS), polyethylene terephthalate (PET), polycarbonate (PC)	Shear-induced polymer alignment	C2C12	20
Semi-synthetic	Self-assembling peptide	N/A	C2C12	17
Natural	Collagen	Shear-induced polymer alignment	C2C12	26
	Collagen	Unidirectional Gold nanowires (GNW)	C2C12, human adipose derived stem cells Sprague–Dawley rats	34
	Gelatin/alginate	Shear-induced cell alignment	C2C12, HUVECs, athymic nude mice	25
	GelMA/alginate	N/A	C2C12	19, 21
	GelMA/alginate	N/A	Human mesenchymal stem cells (hMSC)	22
	GelMA/cellulose	N/A	C2C12	19
	Fibrinogen/gelatin/hyaluronic acid (HA)	Pillars	C2C12	24
Multiple	Polyethylene glycol (PEG)/fibrinogen	Shear-induced polymer alignment	C2C12, subcutaneous implantation in immunocompromised mice	18
	GelMA with polyethylene glycol diacrylate (PEGDA)	N/A	C2C12	19
	Alginate/Pluronic F-127	Shear-induced cell alignment	C2C12	23
	(dECM-MA)/polyvinyl alcohol (PVA)	Shear-induced polymer alignment	C2C12	27

Extrusion printing is promising for muscle tissue engineering, with three-dimensional control and demonstration of several materials able to induce myotube alignment without secondary supporting materials/pillars.^18,23,25,26^ Of particular note is the method developed by Kim *et al.*, whereby shear-induced alignment of PVA occurs in a decellularized extracellular matrix methacrylate (dECM-MA)/PVA mix. Removal of the PVA results in unidirectional grooves and aligns C2C12s.^27^ Extrusion printing of hydrogel materials has its own set of challenges, requiring specific rheological properties to allow extrusion and maintenance of a deposited shape. Further research also needs to investigate the applicability of shear-induced alignment to a more extensive library of extrusion-materials, commonly known as bioinks.

#### 3. Multimodal printing

Several groups have focused on multiscale biofabrication, using macroextrusion printing along with a nanofibrous element for alignment. The combination of these two elements provides complementary mechanical and alignment cues for muscle biofabrication. Yeo *et al.* have iteratively expanded their toolbox; initially, they reported on 3D- printed polycaprolactone (PCL) struts covered with an aligned electrospun polycaprolactone (PCL)/alginate nanofibrous mesh, with cells extrusion printed on top in a polyethylene oxide (PEO)/alginate blend.^28^ Next, they developed a method to individually coat PCL fibers in an electrospun cell-laden nanomesh, extensively characterizing the fiber alignment process.^29^ Recent works in 2019 and 2020 focus on expansion to a collagen/PVA strut, and demonstration of co-seeded human umbilical vein endothelial cells (HUVECs) and C2C12s.^30,31^ Yang *et al.* have 3D-printed PCL and used direct electrospinning writing along with plasma treatment to induce patterning on the fibers.^32^ Lee *et al.* have developed a fibrilization method where polyvinyl alcohol (PVA)/poly lactic-co-glycolic acid (PLGA) is 3D-printed, aligning the PVA with shear. The dissolving of the PVA leaves grooves on the PLGA. Further, dECM-MA is electrospun into fibers and then deposited onto the grooved PLGA in an aligned manner via electrostatic field.^33^ To induce high alignment in extrusion printed collagen, Kim *et al.* added gold nanowires (GNWs) that align during the extrusion printing process. Application of an external electric field provided further alignment of GNWs. The alignment of the nanowires induced a higher degree of myoblast alignment and myotube formation.^34^ Kim *et al.* present a widely adopted measure of printability (Pr) in their data,^34^ an assessment of bioink quality.^51–53^ Multiscale biofabrication demonstrates the power of combining topographical cues with bulk deposition, producing aligned and maturing muscle tissue constructs. As these methods rely on complex fabrication techniques, future focus should be placed on demonstrating their ability to upscale to human-sized constructs. Importantly, the multiscale structures demonstrate that alignment features such as grooves, deposited fibers, or GNWs significantly improve myotube formation.

#### 4. Multimaterial printing

Freeform printing, integrated tissue-organ printing (ITOP), and integrated composite tissue/organ building system (ICBS) techniques are newer extrusion-based printing techniques, relying on supporting materials to create complexity in three-dimensions. Freeform printing leans on a bath of supporting material into which the material of interest is extruded and crosslinked before removing the sacrificial bath. Dixon *et al.* employ freeform fabrication, creating pillars of silk-fibroin where seeded cells can anchor, enhancing alignment.^35^ Human primary skeletal myoblasts were embedded in a collagen, silk fibroin, and Matrigel composite material and pipetted around the silk pillars. Tension induced alignment of cells and self-assembling properties of silk-fibroin improved adhesion between the pillars and materials. Freeform fabrication is a promising fabrication technique for complex 3D structures.^54^ As shown by Dixon *et al.,* tension-based alignment of muscle is achievable using freeform biofabrication. However, this technique is yet to show direct applicability for deposition of cell-laden materials for muscle biofabrication.

ITOP and ICBS utilize several materials (both cell-laden and supporting) in a multimaterial extrusion printing technique.^36–39^ Kang *et al.* in 2016 report human-scale constructs with microchannels for diffusion of nutrients throughout. Supporting materials PCL and Pluronic F-127 have dedicated nozzles in the printer (specific to the heating and printing requirements of each material). Combined gelatin, fibrinogen, HA, glycerol, and C2C12s created a cell-laden bioink, crosslinkable with thrombin. Printed C2C12 mouse myoblasts under tensional support from PCL demonstrated alignment.^36^ Further work in 2018 and 2020 (in the same ITOP system) demonstrate printing of primary human muscle progenitors with a gelatin/HA/glycerol sacrificial material used to create microchannels.^37,38^ Alternatively, Choi *et al.* print PCL pillars to anchor a second material, decellularized skeletal muscle extracellular matrix (mdECM), which crosslinks with heat.^39^ Yeo and Kim extrusion printed PCL support and cell-laden collagen, where homogenous cell-seeding was seen in cell-laden printing compared to post-seeding.^55^ The combination of PCL and hydrogel in these approaches improved alignment and formation of myotubes.

#### 5. 2D and 3D combinations

An alternative technique includes fabrication of cell sheets. This technique involves single-cell layers that are then stacked to form 3D structures. Cell sheets have the benefit of ease of manufacturing but may be limited by the lack of 3D cues during sheet growth. Seeded C2C12s align and form myotubes in a 2D micropatterning technique from Williams *et al.*^56^ Removal of the cell sheets from the substrate allows stacking and layering of the material-free constructs.^56^ Another cell sheet technique uses PCL and Matrigel to form aligned C2C12s.^57^ Laternser *et al.*'s high throughput muscle fabrication for drug testing uses alternate layering of extrusion printing material and inkjet printing of cell-droplets, in a cell sheet-like method.^41^ Bour *et al.* demonstrate a tissue-engineered muscle repair (TEMR) system, where extrusion printing of HA-cell-laden bioink onto both sides of bladder acellular matrix scaffold creates potentially scalable cell sheets.^42^ Although this method is viable for upscaling, true 3D control is lacking.

Miao *et al.* take a different approach to create 3D tissue-engineered muscle. Extrusion printed PVA exhibits a grooved structure due to layering offset. In a sacrificial molding technique, a second material (either agarose, PLA, or soybean oil epoxidized acrylate) is cast into the PVA, which is then dissolved, leaving a grooved 3D scaffold for cell-seeding. The grooves match the alignment of seeded human mesenchymal stem cells (hMSCs) *in vitro*.^40^ This method provides both 3D control and alignment cues; however, only limited materials have demonstrated compatibility. Future work to expand the range of materials into biomimetic hydrogels would be advantageous.

Briefly, other approaches include the development of injectable cell-laden fibronectin ribbons. C2C12s align along the structures and maintain viability on injection.^58^ Quigley *et al.* have demonstrated wet-spinning technique of cell- embedded alginate fibers.^43^ Additionally, Zhu *et al.* demonstrated that implantation of a PCL printed scaffold in the subcutaneous space results in covering of the matrix in ECM.^44^ Removal and subsequent decellularization create a 3D dECM scaffold that can be re-implanted.

Intravital printing occurs directly *in vivo,* removing the need for maturation *in vitro*. Materials are injected and photo-patterned while in the body, allowing direct interaction with the existing vasculature and nervous systems.^45^ Urciulo *et al.* demonstrated that intravital bioprinting of mouse muscle-derived stem cells (MuSCs) into wild-type mice results in *de novo* formation of aligned myofibers. A gelatin and 7-hydroxycourmarin-3-carboxylic acid-based material undergoes cross-linking via coumarin-mediated two-photon cycloaddition. This process is supported by supramolecular interactions that allow close contact of the coumarin photoactive moieties for efficient cross-linking—benefitting from no need for free radical producing photoinitiators. Intravital bioprinting additionally benefits from high resolution features (under 2 *μ*m), which is a characteristic of two-photon polymerization techniques. However, this technology is currently limited to millimeters in depth and size of cross-linking. Intravital printing is also highly reliant on the development of crosslinkable materials with low cytotoxicity. Future work will need to develop methods to up-scale the technology for larger constructs of clinically relevant size for VML and to improve biomimetic materials.

In summary, significant advances in muscle biofabrication techniques have been achieved. Multiscaled and 3D constructs can be fabricated and provide improved control of bulk and micro/nanofeatures. To enhance alignment on the construct, use of either a tensional, shear-induced alignment, or physical surface feature,^59^ biofabrication strategy is recommended. Other techniques of biofabrication include droplet-printing and laser-based. These techniques have been recently reviewed and aside from a brief summary in Fig. 2 are not discussed here. The interested reader is referred to “3D bioprinting for biomedical devices and tissue engineering: a review of recent trends and advances”^59^ for more information.

### B. Bioink properties

An ideal bioink possesses properties that are uniquely relevant to the precise application. Specifically, mechanical strength, rheological properties, porosity, and cell adhesion must be tuned to meet the requirements of both the mode of printing and the encapsulated cells.^60^ Hydrogels can possess characteristics of non-Newtonian fluids and exhibit high levels of nutrient diffusion, making them ideal candidates for bioinks. Ideal bioinks experience shear-thinning and thixotropic properties, liquifying under shear-stresses to facilitate extrusion, and re- gelating quickly to maintain construct integrity.^61–64^ Typically, extrusion-based printing favors high viscosity and thixotropy, while inkjet and light-based methods rely on low viscosity.^60^ Mechanical strength can be improved through further cross-linking mechanisms (photopolymerization,^65,66^ chemical,^67,68^ and enzymatic^69,70^) during or after printing.

We recommend that readers consider the following assessments when reporting extrusion printed 3D constructs: bioink quality (for fabrication), which may include filament and layering assessments,^51,53,71–73^ and assessment of rheological measures of viscosity,^74^ loss tangent,^75^ yield stress,^76^ and shear-thinning.^61–64^

Mechanically, the bioprinted scaffold must possess sufficient strength to withstand gravity and handling during maturation and implantation, yet needs to demonstrate suitable stiffness to facilitate effective tissue regeneration. By tuning hydrogels to increase stiffness, constructs can be endowed with enhanced durability; for example, enhanced proliferation and migration of C2C12 myoblast cells has been demonstrated with controlled stiffness.^77^ However, the degree of stiffness needs to be balanced with cell requirements, with many publications highlighting the relationship between material stiffness and cell behavior.^78–81^ Synthetic polymer bioinks are favorable for their high tunability, but lack binding motifs found in most naturally derived hydrogels. Conversely, natural bioinks exhibit good cell binding and migration, but lower tunability. Typically, natural hydrogels such as collagen I and fibrin have been used as conductive microenvironments for growth and differentiation of skeletal myoblasts. The 3D environment of the hydrogels supports the spreading of muscle cells, and unidirectional alignment of cells through geometric constraints and macroscopic tissue contractions. Although hydrogel-based muscle tissues have been able to comprise aligned and striated myotubes, their contractile forces are limited. The limitation of their contractile forces is noted to insufficient myotube diameter, volume density, and/or level of functional differentiation.^82^ Future works in muscle tissue engineering should consider the interplay of tissue functionality and hydrogel mechanical properties. The effects of material mechanical properties on cellular behaviors are a compelling field, and we point the interested reader to this recent review.^79^

Although mechanical strength is an invaluable property, it must be balanced against hydrogel porosity and density to optimize viability and cell growth. An alternate means to improve strength is via photopolymerization, enzymatic, or chemical cross-linking, lending additional strength to constructs during or after printing. Additionally, this strategy allows for multistage control of physical properties, by inclusion of gels that respond to different cross-linking reagents, thus minimizing viscosity during extrusion (as high viscosities are linked to lower cell viability)^83^ but maximizing strength post-print. Zhu *et al.* demonstrated this technique by cross-linking alginate with CaCl_2_ during printing to act as a scaffold and then cross-linking GelMA; the alginate could then be dissociated to leave the more biocompatible, and now mechanically stable, GelMA for seeding.^22^

A successful bioink will function like the native ECM to orchestrate cell adhesion, migration, proliferation, and differentiation into a cohesive and functional tissue construct. Non-immunogenicity, nontoxicity, a similar degradation rate to native tissue, and nontoxic degradation products are all invaluable considerations when designing a bioink to maximize cytocompatibility and construct viability *in vivo*.

## III. MATURATION OF CONSTRUCTS

### A. Construct architecture

The core goal of 3D skeletal muscle bioprinting is to produce functional tissue constructs that can interact with and integrate into host tissue. Structure determines the function of skeletal muscle; each muscle fiber subunit contains many actin-myosin cross bridges, each producing minute amounts of contractile force that, when organized in a parallel arrangement, produce a much greater net force.^84^ There is substantial evidence that *in vivo* cell and overall tissue organization is influenced by the skeletal muscle ECM, as reviewed by Csapo *et al.*^85^ Hydrogels form a network of fibers, analogous to native ECM fibers, and similarly are capable of supporting tissue development.

This function can be enhanced by incorporating aligned micropores or microchannels into 3D printed scaffolds. Lyophilization is one typical method for forming micropores, which takes advantage of the inherent anisotropic freezing properties of water and leaves behind unidirectional pores following a freeze-drying procedure.^86^ In a type I collagen hydrogel, the size of micropores produced through lyophilization was shown to be tunable, with dependence on freezing temperature, collagen concentration, and incorporation of detergent and acetic acid.^87^ Although many studies have been successful in demonstrating the production of anisotropic hydrogel constructs, there have been few published examples of using lyophilized scaffolds to align myotubes. In one study by Jana *et al.*,^88^ C2C12 myoblasts successfully formed long myotubes with large diameters in a chitosan scaffold. Another study by Velasco-Mallorquí *et al.*^89^ investigated lyophilization of carbon nanotube (CNT)-doped gelatin-microcellulose for myoblast alignment; the resultant constructs were found to be highly aligned, exhibiting suitable nutrient diffusion. Incorporated CNTs also improved construct conductivity, a property implicated in *in vivo* muscle development. Unlike bioprinting, the harsh conditions used to produce micropores in lyophilization exclude the possibility of preseeding, so cells must be seeded post-production. Biofabricated microchannels are an alternative to lyophilization demonstrated to be highly conducive to vascular and neural ingrowth, thus supporting the development of larger muscle constructs. Microchannels can be formed by printing a multicomponent construct, typically composed of a seeded bioink and a sacrificial component; for example, Lee *et al.*^90^ recently demonstrated the use of dissolvable poly-(N-isopropylacrylamide) (PNIPAM) electrospun fibers as a sacrificial element to form microchannels. This study proved successful in treating severe hindlimb ischemia and improving recovery time in wound closure in a mouse model. The method indicates that the success is due to promotion of macrophage invasion and angiogenesis.

Another notable example of microchannel use by Kim *et al.*^37^ utilized three materials: one bioink (gelatin/fibrinogen/HA/glycerol composite) containing human primary muscle progenitor cells (hMPCs), a gelatin- based sacrificing hydrogel, and a poly(ε-caprolactone) (PCL) support structure. Post-print, fibrinogen in the bioink underwent cross-linking before the sacrificial bioink was dissolved out. Seeded hMPCs were shown to form highly aligned myofibers and demonstrated a high cell viability due to improved diffusion capacity. Post-implantation, the construct restored 82% function in a rat tibialis anterior (TA) muscle defect model, with high levels of host vascularization and neuromuscular junction formation at week 8. A follow-up study has since demonstrated that the inclusion of human neural stem cells (hNSCs), at the optimal hMPC:hNSC ratio of 300:1, improves myogenic differentiation, rat TA VML model recovery time, and neuromuscular junction formation.^38^ The team proposed that hNSC-derived myogenic factors enhanced adhesion and differentiation.

### B. Seeding constructs and bioreactors

Construct scaffolds can be seeded at two stages: post-print by immersion in liquid culture, or preprint by inclusion in a bioink. In the former method, microtopographical cues at the surface^49^ have proven capable of aligning and differentiating myoblasts into myotubules. Innate cues from decellularized ECM have demonstrated guided development of complex cardiac tissues^91^ and skeletal muscle.^27,33,39,44^ However, bioinks offer a more homogenous cell distribution when compared to post-print seeding, which can result in localization of cells to the surface,^55^ making bioinks more beneficial for the growth of 3D skeletal muscle constructs. Cell types for muscle engineering have recently been reviewed^5,92–94^ and therefore will not be extensively discussed here. Nevertheless, briefly, tissue-engineered skeletal muscle is primarily composed of stem-progenitor cell lines and is supported by several other cell types: fibroblasts, macrophages, vascular endothelial cells, and motor neurons. Future works should take into account the dynamic signaling between cell types and the importance of studies using human cells.

**FIG. 3.**
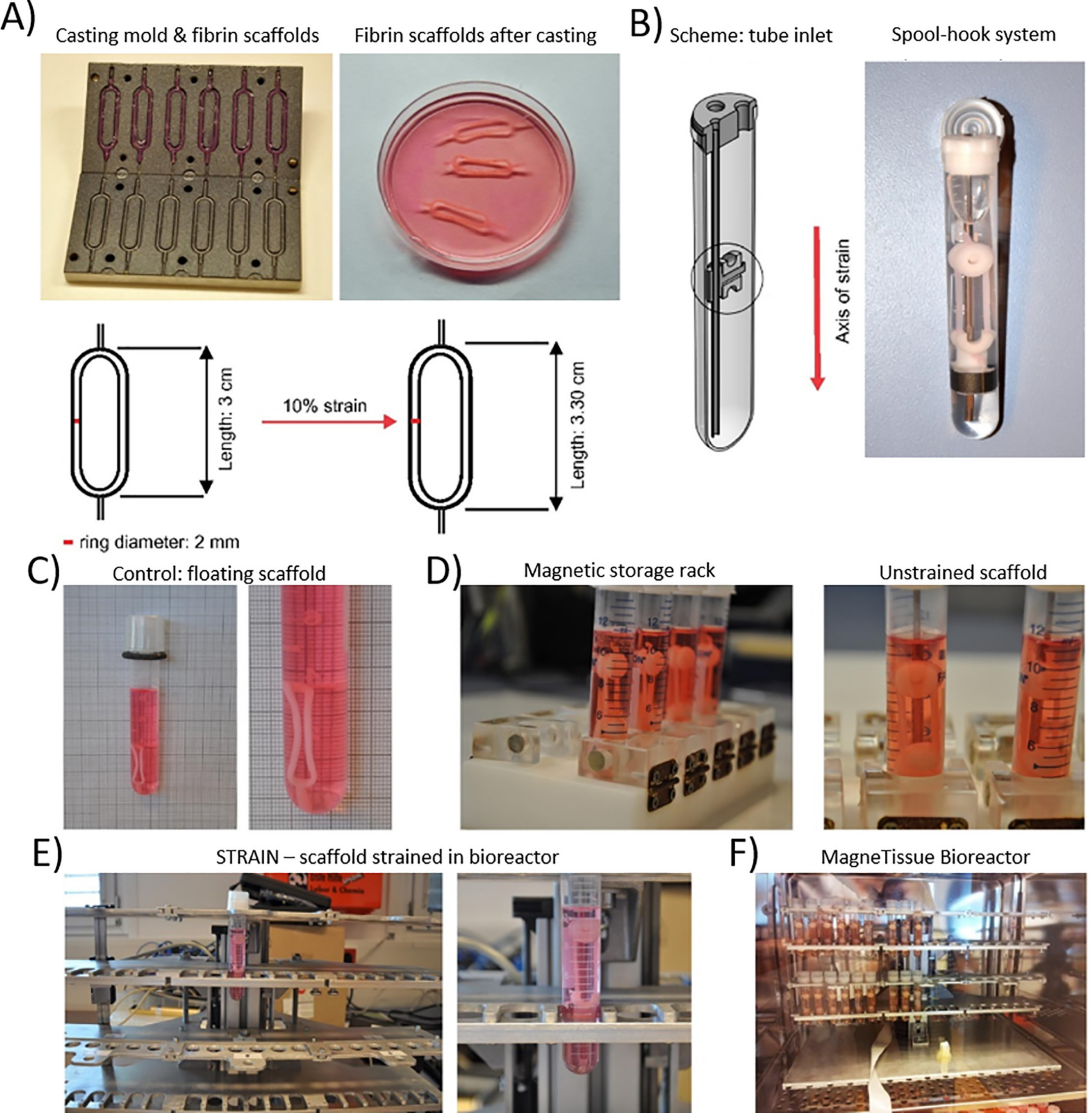
MagneTissue bioreactor. (a) displays the mold used for scaffold preparation, as well as the resulting fibrin scaffold. (b) displays the spool-hook system, which applies mechanical strain to the fibrin scaffold and seeded cells. (c) displays scaffold that is not undergoing mechanical stress, whereas (d) presents the magnetic storage rack, which applies mechanical stress with the spool-hook system during culturing. (e) displays bioreactor after 3 days of culturing. (f) presents the entire MagneTissue system. Reprinted with permission from Heher *et al.*, Acta Biomater. **24**, 251–265 (2015). Copyright 2015 Elsevier.^96^

Bioreactors (Fig. 3) are systems for supporting the expansion of cell and tissue cultures by closely mimicking *in vivo* conditions. A perfect bioreactor would ideally be able to guide the complete development of a skeletal muscle organ including vascular, neural, and tendinous components. At present, uses for bioreactors include expansion of cell culture, and maintenance and development of cells in a printed scaffold. Many bioreactor systems are currently available. Common requirements include suitable nutrient media and control of several key components of niche cellular environments including temperature, pH, and biochemical requirements of cells. Spinner flask, rotating, and perfusion-based bioreactor systems for skeletal muscle cell expansion have been reviewed by Yang and Dong.^95^ Several new bioreactor systems have recently been developed specifically for skeletal muscle tissue, taking advantage of the role that mechanical strain and electrical stimulation play on growth *in vitro*. Static mechanical strain bioreactors such as the MagneTissue, shown in Fig. 3, have been demonstrated to significantly aid in the differentiation and maturation of long myotubes^96^ and with the advent of automation could become a mainstay in skeletal muscle engineering. Similarly, electrical stimulation has proven effective in developing mature adult skeletal muscle phenotypes, demonstrating increased force generation, and potential tunability of slow and fast myosin content.^97,98^

Additionally, bioreactors allow for the culturing of cellular seeded scaffolds prior to implantation, allowing for a semi-mature tissue construct to be implanted into the patient. The first literature recorded attempt was conducted in 2013 where researchers investigated the effect of cellular density upon the maturation of skeletal muscle constructs, concluding it to be a key factor when culturing a construct.^99^ However, significant progress has been made since then, and researchers have shifted their focus to the integration of other structures into mature constructs, such as nerve integration^38^ and vasculature.^94^ Using this approach, researchers have made great strides toward the *ex vivo* maturation of a skeletal muscle construct, but this progress further illustrates the great complexity that *in vivo* structures possess. Further consideration needs to be placed in bioreactor systems capable of supporting these complex cells and structures to allow for the full maturation of a skeletal muscle construct.

### C. Growth mediators and cell selection

Growth mediators are a versatile group of molecules involved with cell adhesion, growth, migration, alignment, and differentiation. Growth mediators can be incorporated into a solid hydrogel scaffold or liquid differentiation media. Laminin 111, an ECM component, is highly conducive to alignment and differentiation when compared to collagens I and IV, potentially due to the affinity of skeletal muscle integrins and dystroglycan receptors for laminin 111.^100^ Laminins can be found in many decellularized ECM bioinks, acting as adhesion molecules during development to allow for migration and maturation.^39^

Fibroblast growth factor (FGF), platelet-derived growth factor (PDGF), hepatocyte growth factor (HGF), and insulin-like growth factor (IGF), reviewed by Syverud *et al.*,^101^ are growth factors currently being explored for skeletal muscle bioengineering applications. FGF and PDGF enhance proliferation of satellite cells, HGF causes myogenic lineage commitment, and IGF has a role in proliferation and differentiation. HGF and FGF have also been shown to impede differentiation, allowing for enhanced proliferation of satellite cells in early stages of construct development.

Paracrine factors released from neurons and glial cells during the formation of Neuromuscular Junctions in muscle progenitor cell coculture can also enhance proliferation and differentiation.^38,102^ Brain-derived neurotrophic factor (BDNF), nerve growth factor (NGF), glial-cell-line-derived neurotrophic factor (GDNF), and neurotrophin-4 and -5 are neurotrophic factors implicated in skeletal muscle fiber development, maintenance, and regeneration,^103^ indicating potential future use in skeletal muscle bioprinting applications. IGF-1 and IGF-2 have also been shown to be involved in muscle development and repair, with roles in proliferation and differentiation during development, and the ability to induce myogenic commitment in placental mesenchymal stem cells.^104^ Further, future studies may incorporate angiogenic factors such as vascular endothelial growth factor, and neurogenic factors such as sonic hedgehog in the development of more complex tissue structures.

Further to growth mediators, cell selection remains a key challenge, with researchers often opting for immortalized cell lines. Although these cells provide some indication of the efficacy of the tested approach, they fail to adequately model the complexity of native tissue regeneration. C2C12 murine cells are routinely employed in tissue regeneration studies, despite inherent limitations associated with their use, including morphology, immortal state, and divergence from gene expression patterns exhibited in freshly harvested primary satellite cells.^105^ Another muscle cell line, MM14, displays a greater likeness to primary satellite cells, yet these still fail to adequately model behave as primary cells, owing to a dependence on exogenous FGF stimulation during G1 transition to avoid terminal differentiation.^106^ For further details on the importance of cell selection, we direct the interested reader to this excellent review.^105^

### D. Vascularization

**FIG. 4.**
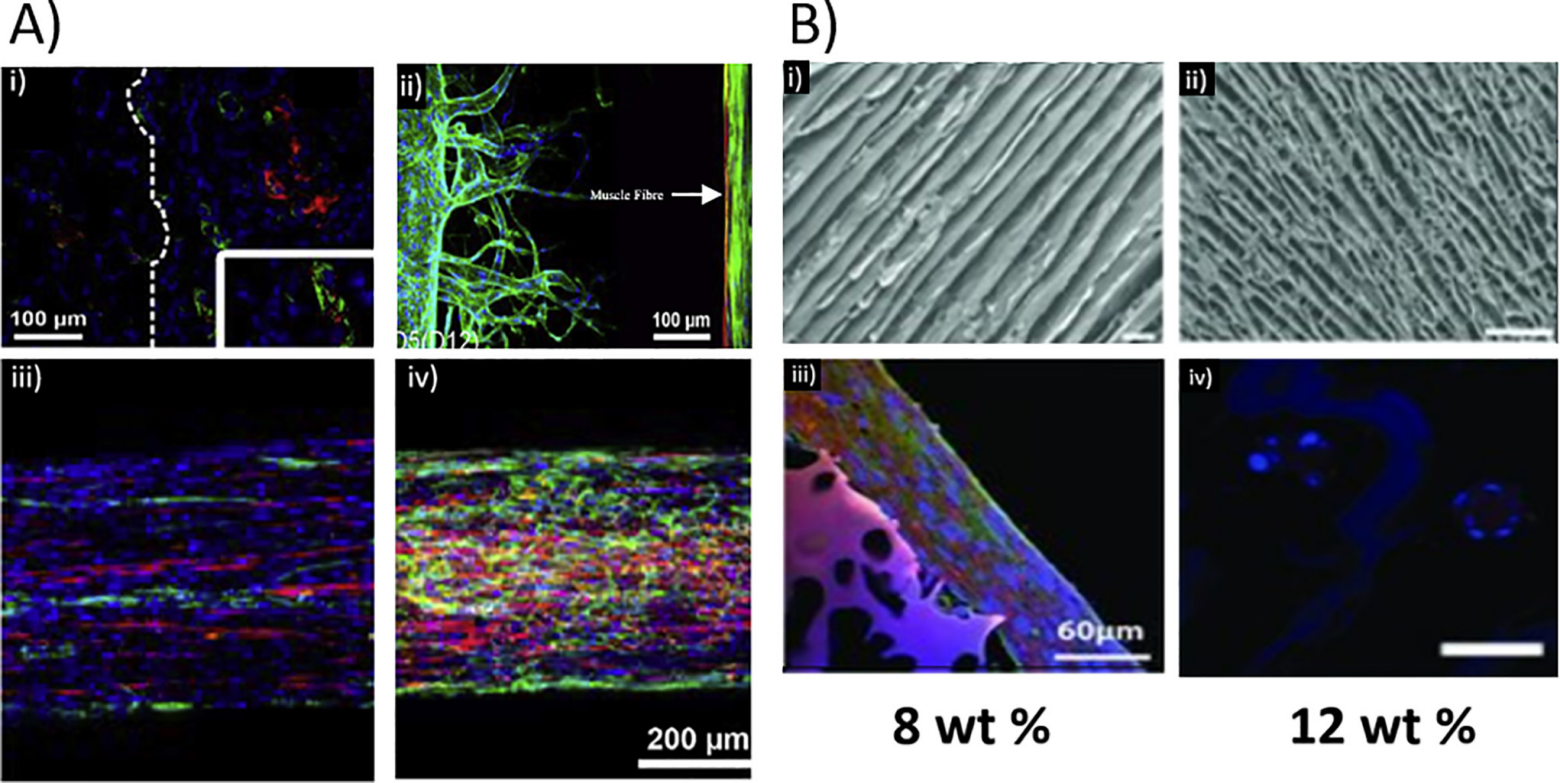
Skeletal muscle engineering strategies. (a) Development of vascularized skeletal muscle tissue using (i) decellularized scaffold stained for angiogenic response after implantation (15 days), alfa-SMA (green), vWF (red), and DAPI (blue). (ii) Vascularized muscle bundle engineering highlighting the formation of vasculature channels and showing sprouting of HUVECs toward muscle fiber and formation of capillary networks (muscle fiber indicated by arrow). Muscle bundle and microvasculature formed using a hydrogel casting and injection method, as reported by Osaki *et al.* Sample stained using Phalloidin (green), DAPI (blue), and α-actinin (red). (iii) and (iv) Printing of decellularized vascularized muscle using (iii) mixed population of human skeletal myoblasts (hSKM cells) and HUVECs, and (iv) coaxial printing with decellularized vascular tissue containing HUVECs on the shell of the fiber and skeletal muscle decellularized tissue containing hSKM cells localized to the core. Samples stained using CD31 (green), DAPI (blue), and MHC (red). (b) The role of architecture in maturation of myotubules. Top: SEM images of chitosan scaffolds prepared from solutions of (i) 8% or (ii) 12% chitosan concentrations. Bottom: images were obtained by immunocytostaining, MHC staining, and nuclei staining upon cells grown on the (iii) 8% and (iv) 12% chitosan scaffolds. Scale bars: (i) and (ii) 150, (iii) 60, and (iv) 50 *μ*m. (a) (i) Reproduced with permission from Alvarez Fallas *et al.*, Int. J. Mol. Sci. **19**(5), 1319 (2018). Copyright 2018 Authors, licensed under a Creative Commons Attribution (CC BY) license.^126^ (ii) Reproduced with permission from Osaki *et al.*, Biomaterials **156**, 65–76 (2018). Copyright 2018 Authors, licensed under a CC-BY-NC-ND 4.0 license.^127^ (iii) and (iv) Reprinted with permission from Choi *et al.*, Biomaterials **206**, 160–169 (2019). Copyright 2019 Elsevier.^145^ (b) Adapted with permission from Jana *et al.*, Adv. Healthcare Mater. **2**(4), 557–561. Copyright 2013 Wiley.^88^

The delivery of oxygen and nutrients, as well as the removal of metabolic waste products, is vital for the function of skeletal muscle. Issues with skeletal muscle construct vascularization have hindered progress to the clinical setting. Engineered muscle constructs cannot exceed ∼0.4 mm in thickness due to insufficient oxygen diffusion for maintaining cell viability, as oxygen diffusion distance is ∼200 *μ*m from the construct edge.^107–109^ Consequently, the effective vascularization of tissue engineered constructs is a significant issue; limited by advances in vascularization strategies, and as such, is a major focus of tissue engineering.^110–112^ Two approaches to vascularization of engineered tissues are angiogenesis, where the in-growth of new blood vessels from the surrounding tissue is encouraged, and inosculation, where defined regions within the constructs can be directly incorporated during the biofabrication process, matured into functional blood vessels *in vitro*, and connected to prefabricated perfusable vasculature.^113,114^ As shown in Fig. 4, angiogenesis has been induced by cytokine, gene and cell-based approaches,^115–117^ and material properties such as scaffold density^118,119^ and stiffness.^120,121^

In studies of tissue engineered muscle, several studies demonstrated vascularization via angiogenesis-inducing or inosculation methods. *In vivo* studies of the ITOP fabrication method indicated successful inosculation. Implantation into nude rats^36^ and Rowett Nude (RNU) rats^37^ of ITOP fabricated, cell-laden constructs with vascular- like microchannels demonstrated inosculation, as indicated by von Willebrand Factor (vWF) staining. Inosculation is a relatively quick method for suitable provision of nutrients to embedded cells. However, as one study shows, over time decreases in mechanical strength of channels may render them un-perfusable.^122^ Fabricated vasculature will need to be compared to angiogenic vasculature to establish the long-term viability of each method.^127^ Angiogenic methods of vascularization can be slow to form, especially in clinically relevant sized constructs. Intravital printed muscle construct demonstrated angiogenesis after 10 days in C57BL/6J mice,^38^ indicating the developed gelatin-based material supports in-growth from the surrounding tissues.

Angiogenesis occurred in a study by Juhas *et al.* who implanted engineered skeletal muscle into mice.^123^ Their results showed angiogenesis from host blood vessels, despite no evidence of vascular cells at the time of implantation. By the 14th day of their study, the implanted muscle with no vascular cells displayed equal vascularization to implants with preformed vascular structures. These results show that angiogenic vessel ingrowth is sufficient to support the *in vivo* survival and function of small (1 mm diameter) avascular engineered muscles and indicate that the scaffolding material of Matrigel and fibrinogen is suitable for *in vivo* angiogenesis.^123^ An alternative approach to promote angiogenesis is the co-seeding of HUVECs with C2C12s, which has demonstrated microvessel formation *in vitro.*^31^ Co-axial printing of two bioinks, one containing endothelial cells and a second muscle cells have shown some success in generating prevascularised constructs, albeit on a relatively small scale; further testing is required to ensure that they are functional and robust vessels.^145^

However, angiogenesis is slow and can take several weeks to develop sufficient vasculature in substantial constructs, ultimately resulting in necrosis in the center.^124,125^ An approach that can be used as a supplementary to angiogenic vasculature is oxygen-delivering technologies.^108^ Seyedmahmou *et al.* demonstrated that the addition of oxygen-generating calcium peroxide particles in extrusion printed gelMA/alginate increased the metabolic activity of embedded C2C12s.^21^ However, more extensive studies need to be undertaken to assess the long-term effects of oxygen-delivering techniques on cells. Future studies should consider that a combination of biofabricated microchannels for inosculation along with angiogenic factors and possibly oxygen-delivering techniques will be needed to create clinically relevant engineered tissues for VML.

### E. Innervation

**FIG. 5.**
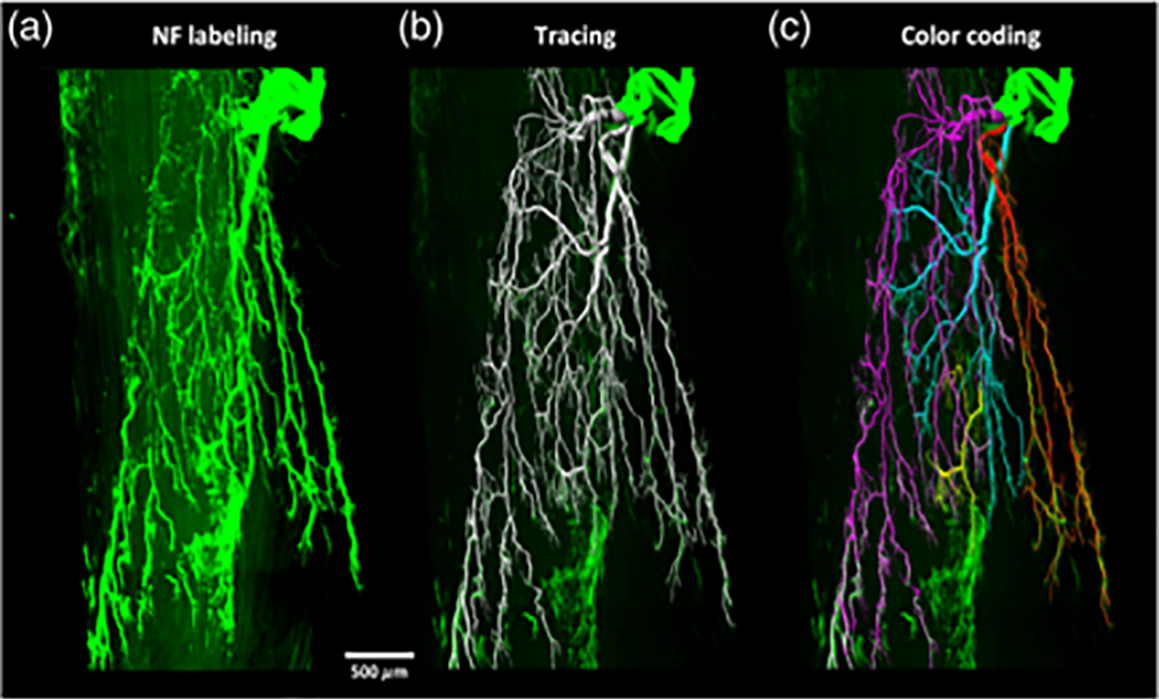
Three-dimensional visualization of motor nerve fibers within an adult mouse tibialis anterior using (a) light sheet fluorescence microscopy (LSFM), (b) computerized segmentation, and (c) color coding of the different nerve branches. Reproduced with permission from Li *et al.*, Neurophotonics **7**(1), 015003 (2020). Copyright 2020 Authors, licensed under a Creative Commons Attribution (CC BY) license.^13^

Neural innervation is the process by which motor nerves interact with muscle as shown in Fig. 5.^13^ This process is therefore required for the restoration of muscle function and the prevention of muscular atrophy, as seen in nervous system conditions such as motor neuron diseases (MND).^128^ Motor units, composed of a motor neuron and the group of myofibers it innervates, have been demonstrated to undergo physiological changes due to age-related progressive muscular atrophy.^129^ Therefore, it is imperative to restore motor neurons and neuromuscular junctions post-implantation within the host. Neuromuscular junctions are highly specialized structures involving neurotransmitter diffusion across “synaptic gutters,” spaces between motor neurons and myofiber groups.^130,143^ Neuromuscular junctions are intricate and highly specialized, allowing for reliable signal transmission between muscles and nerves. Biofabrication of innervated structures is a developing field, with limited studies demonstrating functional innervation.^131^ Bioprinting holds promise for the development of highly complex structures, such as those seen at the neuromuscular junction, due to the precise placement of multiple cell types in cohort. Human primary myoblasts and human induced neural stem cells combined in an *in vitro* freeform fabrication of silk-fibroin, and collagen/Matrigel hydrogel demonstrate the formation of neuromuscular junctions as indicated by formation of acetylcholine receptors (alpha-bungarotoxin stain).^35^ Kang *et al.* ectopically implanted ITOP fabricated cell-laden PCL/gelatin/fibrinogen/HA/glycerol into nude rats and demonstrated the formation of neural integration. After 4 weeks, positive markers included neurofilament, alpha-bungarotoxin indicating the formation of neural junctions.^36^ Further work from the ITOP group fabricated constructs laden with primary human muscle progenitors. They demonstrated the beginning of neural integration and 85% muscle force regained in Rowett Nude (RNU) rat muscle defect model after 8 weeks (30%–40% tibialis anterior loss).^37^ Moreover, use of primary human muscle progenitors with human neural stem cells in the same RNU rat muscle defect model demonstrated full restoration of muscle force and neuromuscular junction formation after 8 weeks.^38^ Neural innervation has been achieved in mice where a muscle construct is implanted near established native nerves, but this has not been investigated in larger organisms and requires further study to determine the mechanisms of this phenomenon. Additionally, future work should focus on models with immunocompetency. Neural integration is imperative for the restoration of functional tissues.^128^ Biofabrication holds promise to create the complex neuromuscular junctions with the ability to precisely place multiple cells types in tandem. However, limited studies characterize or even include neural cell types in their biofabricated muscle tissues. Future works should continue to take advantage of biofabrication to promote innervation for functional tissue formation for improved clinical outcomes.^131,132^

### F. Myotubule elongation and maturation

**FIG. 6.**
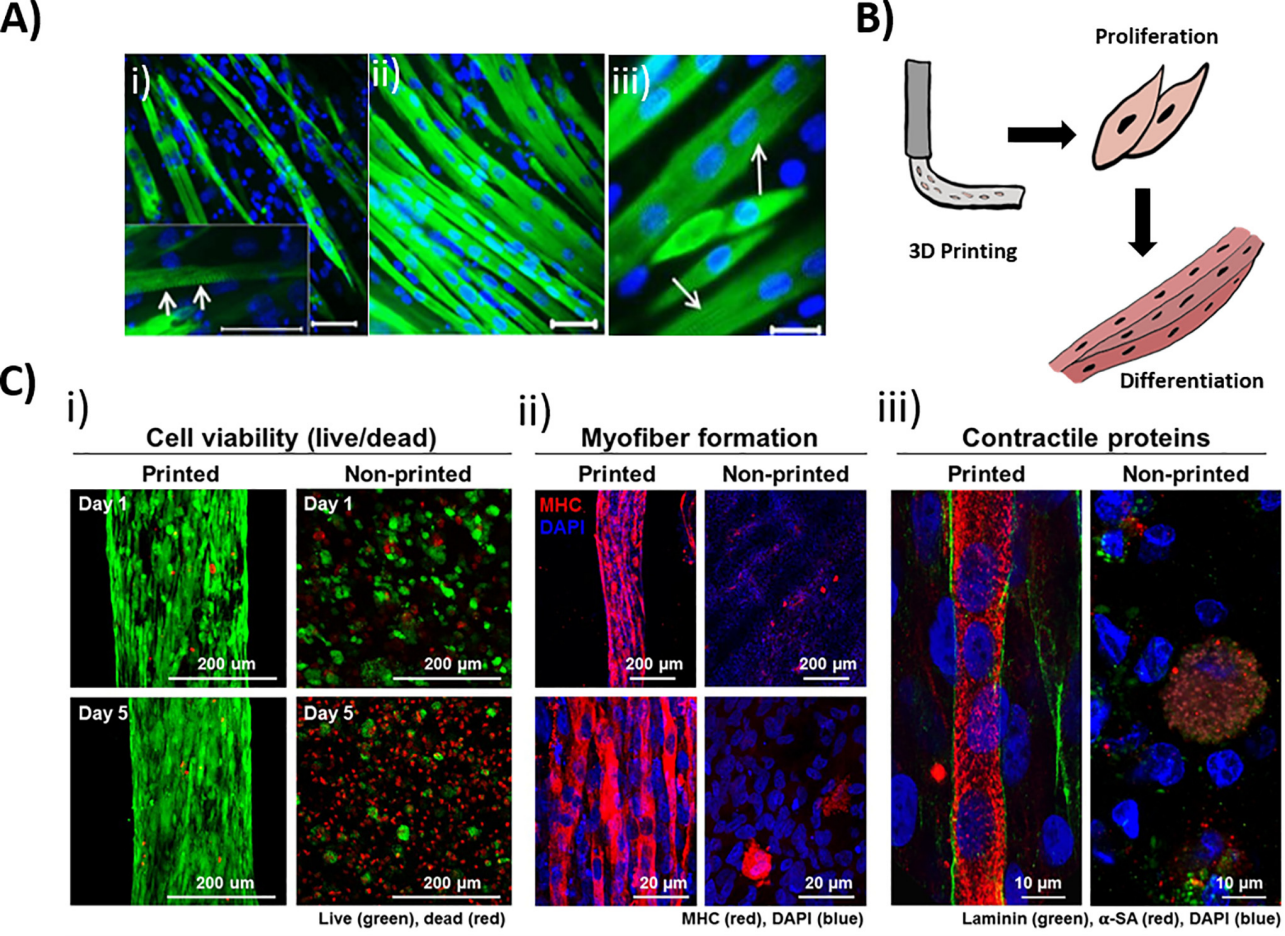
Maturation of tissue engineered muscle. (a) Indications of maturity in (i) non-printed 3D culture of C2C12 s in muscle dECM demonstrates (ii) multinucleated myotubes by day 3 (scale bar 50 *μ*m) and (iii) the beginning of striations by day 6 (arrows) (scale bar 25 *μ*m). (b) Graphic of extrusion printing biofabrication process. (c) Comparison of 3D culture and 3D fabrication (ITOP system) of primary human muscle progenitors in a gelatin/HA/fibrinogen/glycerol bioink, showing alignment enabled through printing-induced placement and tensional forces from PCL supporting material. (i) Viability in printed and non-printed materials at 1 and 5 days. (ii) Staining of maturation markers, where myosin heavy chain and DAPI staining demonstrate multinucleated myofibers after 7 days of differentiation. (iii) α-sarcomeric actin, laminin, and DAPI staining of deposited ECM protein and striations of contractile protein. (a) Adapted and reproduced with permission from Chaturvedi *et al.*, PLoS One **10**(6), e0127675 (2015). Copyright 2015 Authors, licensed under a Creative Commons Attribution (CC BY) license.^12^ (c) Reproduced with permission from Kim *et al.*, Sci. Rep. **8**(1), 12307 (2018). Copyright 2018 Authors, licensed under a Creative Commons Attribution (CC BY) license.^37^

Following cellular expansion, seeding, proliferation, and migration, myoblasts become committed to the myogenic lineage and proceed to the processes of maturation (Fig. 6). Typical methods for inducing myotube formation involve aligning myoblasts so that fusion occurs unidirectionally by utilizing micropatterning and incorporating microchannels.^37,38,47–49^ Another additional method of note involves extrusion of semi-differentiated myoblasts, which, having gained partial-elongation, demonstrate anisotropic behaviors in response to shear stresses in the nozzle, aligning in the direction of the print and developing highly aligned tissues.^18,23,25,26,133^ These methods are successful, as myoblasts can recognize and adhere to colinearly aligned neighboring cells, fuzing to form multinucleated myotubes with centrally located nuclei and some myofibrils. Further fusion of myotubes and increased actin and myosin production leads to the formation of myofibers with large bundles of myofibrils and peripherally located nuclei.^134,135^ In tissue engineered muscle, markers of maturation are often reported, commonly including DAPI nuclei staining to visualize multinucleated fibers, and indicators of protein formation such as myosin heavy chain, F-actin, and α-sarcomeric actinin. The correct spatial and temporal arrangement, and fusion of myoblasts during development and tissue repair, is controlled by a multitude of transcription regulators,^136^ and complex interplay between the environment and other cell types; however, these factors are beyond the scope of this review.

## IV. IMPLANTATION AND RESTORATION OF FUNCTION

### A. Immunological considerations and degradation

To date, research into the *in vivo* immunological responses of bioprinted scaffolds for VML applications has been limited. Many *in vivo* models exist to study muscle injury; for example, where immunocompetent mice are subjected to volumetric muscle loss and ischemic injury.^137^ Some reports have shown side by side immunological responses leading to enhanced regeneration from processed^2^ or decellularized tissue grafts^138^ in immunocompromised animals. However, many studies involving 3D fabricated constructs have, by their nature, involved immunocompromised rodents, which may affect conclusions about the foreign body response and construct degradation. Of the studies in immunocompetent models, alignment, anti-inflammatory, and natural material strategies have demonstrated decreased foreign body response. Reduced fibrosis was demonstrated on the implantation of aligned biofabricated constructs into Sprague–Dawley rats compared to nonaligned samples by Kim *et al.* After 8 weeks, substantial myofiber maturation had occurred with aligned constructs, indicating alignment contributes to decreased fibrosis. Additionally, the anti-inflammatory effects of embedded gold nanowires indicate a beneficial environment for regeneration.^34^ Any immunological response initiated against constructs is likely to be targeting hydrogel components rather than cellular components, as these are sourced from the patient's own tissue. Despite being one of the earliest stages in construct production, gel choice is also one of the most critical in successful skeletal muscle bioprinting. PLGA/PLA cell-laden structures implanted into mice type C57BL/10ScSn and C57BL/10ScSn-Dmd^mdx^ (Duchenne's muscular dystrophy) demonstrated significant foreign body response compared to alginate-based structures.^43^

After 12 weeks implantation, alginate constructs had begun degradation *in vivo*, whereas PLGA/PLA remained intact. Studies in mouse models have noted that ECM-derived hydrogels have a lower propensity for triggering immune activation than their synthetic counterparts,^139^ but all surgery results in some form of inflammation as part of the natural healing process.^140^ In the study of intravital printing, no difference in histology, apoptosis, or macrophage infiltration was seen between the phosphate buffered saline control and the gelatin-based material in C57BL/6J mice,^45^ indicating that the gelatin-based material does not provoke significant foreign body response. An unfortunate attribute of some synthetic gels is poorer degradation responses when compared to naturally derived gels, potentially due to a lack of binding motifs on which native cells can act, requiring synthetic gels to be functionalized with bioactive binding motifs.^76,141^ To overcome the immune issues posed by synthetic gels while maintaining desirable material properties, emerging classes of bioinks incorporate the benefits of synthetic hydrogels, namely, tunability and rapid chemical synthesis, along with the cytocompatibility, favorable adhesion, and degradation rates of naturally derived hydrogels. One such class is the peptide hydrogel, recently demonstrated to display minimal immune activation in an immunocompetent rat model, while possessing tunability in amino acid sequence as well as through extrinsic factors such as pH, temperature, and salt concentration, when embedded as subdermal disks.^142^ Future studies should focus on the use of materials with high immunocompatibility and report data within immunocompetent models.

### B. Current successes

Whilst there have been numerous *ex vivo* examples of muscle bioprinting,^21,38,39,144^ there have been a number of *in vivo* successes for 3D bioprinting: for example, researchers have successfully implanted engineered skeletal muscle into rats.^37^ The rats underwent a surgical procedure where 30%–40% of the tibialis anterior muscle was removed and replaced with a human skeletal muscle construct laden with human muscle progenitor cells. Kim *et al.* observed 82% muscle functionality restored.^37^ While there are obstacles remaining to engineer tissue constructs capable of addressing volumetric muscle loss within humans, this success is promising. Refer to Table II for more successes.

**TABLE II.** Summary of muscle bioprinting successes.

Article title	Material/s	Successes	Challenges	Reference
3D bioprinted human skeletal muscle constructs for muscle function restoration	PCL, gelatin, fibrinogen, HA, glycerol	Successful implantation of printed construct into rodent model, achieving 82% functional recovery.	Rodent model was immunocompromised, and therefore further study is required into inflammatory and immune responses.	37
Three-dimensional bioprinting of functional skeletal muscle tissue using gelatin methacryloyl- alginate bioinks	GelMA alginate Calcium Peroxide	GelMA gel was capable of cross-linking while maintaining cell viability and muscle tissue formation. Bioink was further improved through the addition of oxygen-generating particles, increasing the metabolic activity of cells.	The addition of oxygen releasing particles in GelMA—alginate bioinks created calcium chloride ions, stimulating cross-linking, and forming a dense gel with cells only capable of surviving one day of culture. Further studies are required into the effects of oxygen releasing particles within the body or in other constructs.	21
Three-dimensionally printed biological machines powered by skeletal muscle		Synthesized functional muscle constructs which responded to electrical stimulation, allowing for directed force generation and motion.	Further study into the integration of vascular networks and nerves is required for the synthesis of larger constructs and *in vivo* implantation.	143
3D cell printing of functional skeletal muscle constructs using skeletal muscle-derived bioink	Skeletal muscle dECM PCL	Successfully decellularized and printed constructs from skeletal muscle-derived ECM bioink. The resulting construct retained receptors and adhesion factors.	Requires living tissue to begin with, cannot yet be synthesized *ex vivo*.	39
Neural cell integration into 3D bioprinted skeletal muscle constructs accelerates restoration of muscle function	PCL, gelatin, fibrinogen, HA, glycerol	Integration of nerve cells into bioprinted muscle construct improved cellular differentiation and long-term survival as well as facilitated the formation of neuromuscular junctions.	Study was performed in an immunocompromised rodent model; therefore, more study is required into immunological effects.	38
Biohybrid robot with skeletal muscle tissue covered with a collagen structure for moving in air		Successful development of collagen encapsulated skeletal muscle construct, termed as a “biohybrid robot” capable of motion and object manipulation.	Construct was small in scale, negating the requirement for adequate vascularization.	144

## V. FUTURE CHALLENGES AND OPPORTUNITIES

The last decade has seen bioprinting research explode, with an exponential increase in the technology's potential applications, especially for soft tissues such as skeletal muscle. Despite this, it is still largely in its infancy, with a multitude of limiting factors delaying its progression to clinical applications. These include limited control over hydrogel architecture, a limited understanding of relevant factors and processes involved in human skeletal muscle development and regeneration, and a lack of *in vivo* human studies or *in vitro* studies with human tissues.

The hydrogel portion of bioinks must provide structural support, adhesion, and architectural cues for migration and differentiation, which are naturally provided by the ECM. Although countless studies have demonstrated some success in hydrogel-mediated cell growth, complex tissues may require more finely tuned construct nanostructure. This is due to the support of hydrogels being provided through random, hydrodynamically self-assembling fibrous networks, in contrast to the ECM, which is laid out and restructured by host cells, such as fibroblasts, to suit the requirements of the given tissue. A higher degree of control over this property of bioinks could allow greater influence on the alignment and functional maturation of skeletal muscle. This has largely been achieved with micropatterning, microchannels, and anisotropic shear force myoblast alignment, which are all limited by scalability and cell viability considerations. Improving our selection of bioink hydrogel components would increase our control over gel properties and architecture at the nanoscale level, given the varied nanoscale topographies observed via electron microscopy in currently used hydrogels.

While naturally derived bioinks currently offer the best viability and adhesion, their non-homogeneous nature and low tunability limit their usefulness in advanced bioengineering applications. Synthetic bioinks possess much greater potential for physiological and mechanical tunability, which could expand the array of bioink applications, creating more specialized cell construct environments. A greater understanding of the physiological and mechanical roles of ECM in tissue development and repair would facilitate the development of more intelligently designed and advanced bioinks. Other native mediators implicated in myofiber development should also be further investigated, including paracrine and contact-dependent signaling, and interactions between myoblast, satellite cell, basal lamina, and neural and angiogenic cells. Similarly, a greater understanding of the initiating factors of angiogenesis and neural ingrowth would support future upscaling to more physiologically patient-similar animal models of VML. Exploiting endogenous signals would lessen our reliance on current cytodamaging methods and could prompt new investigations in related fields, for the treatment of muscular dystrophies and other muscle-wasting disorders.

Another major hurdle to the clinical utility of this technology is the, to date, limited number of human trials. Most studies of skeletal muscle constructs have incorporated C2C12 immortalized murine myoblasts; though a useful tool, these could never be used for human *in vivo* constructs, and may not accurately demonstrate human cell line behavior. This reliance on model systems will likely lead to false assumptions about the nature and requirements of skeletal muscle constructs. Further studies on human cell lines, and specifically, the use of human satellite cells, must therefore be conducted to improve the current scope of research. Further, a key requirement for methods for expanding human skeletal muscle cells in culture must be improved. These techniques require high cell densities, and current cell expansion methods are slow and inefficient, and may require larger, more destructive biopsies to be taken to reach the required cell construct density. Long cell expansion times are also unfavorable, allowing more time for fibrotic tissue remodeling and muscle atrophy to take place in VML patients.

Further to the challenge of facilitating angiogenesis, innervation, and maturation, achieving high cell density remains a limitation, with a trade-off between bioink structure and high cell content a significant challenge. Due to ability to spontaneously form adult cell types, stem cells are commonly investigated as potential resources for cell-based therapy. Stem cells are regarded widely for the regeneration of tissues in several medical applications. Currently, the main sources of stem cells include embryonic, mesenchymal, and induced pluripotent.^146^ Stem cells are typically found in the skin, brain, liver, bone marrow, skeletal muscle, and blood. Typically, tissues from biopsies during surgery are discarded, where cells can be obtained and isolated for research and related activities. Although it is common to retrieve cells during biopsies, other specialized embryonic modalities include somatic cell nuclear transfer, arrested embryos, single cell embryo biopsy, and reprogramming somatic cells.^147^ Overall, these stem cell sources provide sufficient modes of examining patient-like environment for research purposes; however, it should be noted that these sources are usually insufficient, and technologies for the large scale *ex vivo* expansion of stem cells are under investigation. Human pluripotent stem cell culture is a significant area of research outside the scope of this work, and we direct the interested reader to the following review.^148^

Finally, there are technological barriers: mainly surrounding the resolution of bioprinters and the effective control of multibioink deposition. Higher resolution can be obtained with light-based hydrogel curing methods, but with the caveat that currently only a single bioink can be used during printing. While multiple bioinks, containing several cell lineages, can be deposited by extrusion-based bioprinters, but at lower resolution. Many studies have looked at combining these techniques to offset their respective negatives, and it can be expected that as research and current technology advance, these issues can be overcome, leading to more advanced methodologies with the potential to translate to clinical application.

## Data Availability

Data sharing is not applicable to this article as no new data were created or analyzed in this study.
